# Incidence and time trends of drug‐induced parkinsonism: A 30‐year population‐based study

**DOI:** 10.1002/mds.26839

**Published:** 2016-10-25

**Authors:** Rodolfo Savica, Brandon R. Grossardt, James H. Bower, J. Eric Ahlskog, Michelle M. Mielke, Walter A. Rocca

**Affiliations:** ^1^ Department of NeurologyMayo ClinicRochesterMinnesotaUSA; ^2^ Division of Epidemiology, Department of Health Sciences ResearchMayo ClinicRochesterMinnesotaUSA; ^3^ Division of Biomedical Statistics and Informatics, Department of Health Sciences ResearchMayo ClinicRochesterMNUSA

**Keywords:** parkinsonism, drug‐induced parkinsonism, incidence, sex differences, time trends

## Abstract

**Background:**

Epidemiological studies of drug‐induced parkinsonism remain limited.

**Objectives:**

To investigate the incidence and time trends of drug‐induced parkinsonism over 30 years in a geographically defined American population.

**Methods:**

We used the medical records‐linkage system of the Rochester Epidemiology Project to identify all persons in Olmsted County, Minnesota, who received a screening diagnostic code for parkinsonism from 1976 through 2005. A movement disorders specialist reviewed the complete medical records of each person to confirm the presence of drug‐induced parkinsonism associated with dopamine‐blocking or dopamine‐depleting medications.

**Results:**

Among 906 incident cases of parkinsonism from 1976 to 2005, 108 persons had drug‐induced parkinsonism (11.9%). The average annual incidence rate of drug‐induced parkinsonism was 3.3 per 100,000 person‐years, was higher in women, and increased with older age. Drug‐induced parkinsonism was the fifth‐most common type of parkinsonism overall; however, it was the most common type among persons younger than age 40 years. Typical antipsychotic drugs were the most common class of drugs associated with parkinsonism, whereas atypical antipsychotic drugs were rarely involved. The incidence rate of drug‐induced parkinsonism decreased 32.0% per decade (relative risk = 0.68; 95% confidence interval: 0.49–0.94) and 68.6% over the 30 years of the study. The decrease was similar in men (65.2%) and women (69.4%); however, the trend was significant only in women.

**Conclusions:**

The incidence of drug‐induced parkinsonism increased with older age and was higher in women at all ages. Typical antipsychotic drugs were the most common cause. The incidence of drug‐induced parkinsonism decreased over the 30 years of the study because of changes in drug use. © 2016 The Authors. Movement Disorders published by Wiley Periodicals, Inc. on behalf of International Parkinson and Movement Disorder Society.

Parkinsonism is a neurodegenerative syndrome that is pathologically characterized by the degeneration of multiple anatomical structures within the brain. In particular, the progressive protein deposition of alpha‐synuclein or tau in the brain may cause a number of different parkinsonism syndromes.^1^, ^2^ The most common subtype of parkinsonism is Parkinson's disease (PD), with an incidence rate of 14.2 per 100,000 person‐years.^3^ However, the presence of a significant degeneration is not always needed to cause parkinsonism. Parkinsonism may also be caused by exposure to drugs or toxic agents that deplete the dopaminergic system and provoke a syndrome that closely resembles PD. Drug‐induced parkinsonism (DIP) has been reported in the medical literature after the introduction on the market of dopamine receptor blockers for treatment of schizophrenia.^4^ However, in more recent years, the use of atypical antipsychotics may have reduced the risk of DIP because of the weaker effects on dopamine of these new drugs.^5^ Although other classes of drugs (e.g., antidepressants or immunosuppressants) have uncommonly been associated with the development of parkinsonism, antidopaminergic medications appear to account for nearly all cases of DIP.^6^ In this study, we investigated the incidence and time trends of DIP by age, sex, and clinical characteristics in a geographically defined population over a 30‐year period from 1976 to 2005.

## Materials and Methods

### Case Ascertainment

Extensive details about case ascertainment were reported elsewhere.^3^, ^7^, ^8^, ^9^, ^10^ Briefly, we ascertained cases of parkinsonism through the records‐linkage system of the Rochester Epidemiology Project (REP). This system provides the infrastructure for indexing and linking essentially all medical information of the county population.^11^, ^12^, ^13^, ^14^ All medical diagnoses, surgical interventions, and other procedures are abstracted and entered into computerized indexes using the Hospital Adaptation of the International Classification of Diseases, Eighth Revision^15^ or the International Classification of Diseases, Ninth Revision.^16^

We ascertained potential cases of parkinsonism using a computerized screening phase and a subsequent clinical confirmation phase, as described in the original reports.^3^, ^7^, ^8^, ^9^, ^10^ The complete medical records of all persons who received at least one of the screening diagnostic codes from 1976 through 2005 were reviewed by a movement disorders specialist using a specifically designed abstracting form (J.H.B. for the years 1976–1990; R.S. for the years 1991–2005). In addition, we also reviewed the records for all persons who received at least one of the screening diagnostic codes in the years 2006–2010. This extended period of capture ensured that patients who came to clinical attention up to 5 years after the study period were appropriately counted as incident cases if the onset of symptoms had occurred during the study period (lag time between onset of symptoms and clinical diagnosis).

The movement disorders specialist defined the year of onset of parkinsonism and the type of parkinsonism using specified diagnostic criteria and following a manual of instructions.^8^, ^17^, ^18^, ^19^ To be included in our study, persons were required to reside in Olmsted County at the time of onset of parkinsonian symptoms. We excluded persons who denied authorization to use their medical records for research.^11^ All study procedures and ethical aspects of the study were approved by the institutional review boards of Mayo Clinic and Olmsted Medical Center.

### Diagnostic Criteria

Our diagnostic criteria included two steps: the definition of parkinsonism as a syndrome and the definition of the type of parkinsonism within the syndrome. Parkinsonism was defined as the presence of at least two of four cardinal signs: rest tremor, bradykinesia, rigidity, and impaired postural reflexes.^3^, ^8^ DIP was defined as parkinsonism with the following three characteristics: (1) symptom onset within 6 months of treatment with dopamine‐blocking or dopamine‐depleting drugs; (2) no parkinsonism symptoms before treatment; and (3) resolution of symptoms within 6 months of withdrawal of treatment (for patients who never discontinued treatment, criteria 1 and 2 were sufficient for inclusion).^8^ We limited the study to only dopamine‐active drugs for three reasons: (1) Parkinsonism linked to other drug classes appears to be extremely rare; (2) antidopaminergic drugs have a plausible mechanism for inducing parkinsonism; and (3) individual cases of parkinsonism associated with other drugs would be much more likely to have alternative causes and would be more difficult to adjudicate.

### Reliability and Validity of Diagnosis and Classification

The case‐finding procedures for parkinsonism were valid and reliable as described more extensively elsewhere.^3^, ^8^ In brief, an independent records review by the two movement disorders specialists who applied the same diagnostic criteria (J.H.B. and R.S.) showed 90.0% agreement on the presence of parkinsonism and 70.0% agreement on the exclusion of parkinsonism (kappa = 0.60; 95% confidence interval [CI]: 0.31–0.89; sample classified by R.S. as 30 patients with parkinsonism and 10 persons free of parkinsonism from the 1991–2005 time interval).^3^ In general, agreement for the year of onset of parkinsonism was also high (intraclass correlation coefficient ICC = 0.85; 95% CI: 0.77–0.92).^3^

### Statistical Analysis

All persons who met criteria for parkinsonism and were residents of Olmsted County at the time of symptom onset between January 1, 1976 and December 31, 2005 (30 years) were included as incident patients. We calculated incidence rates using incident patients as the numerator and population counts from the REP Census as the denominator.^11^ Consistent with previous studies,^3^, ^8^ the denominator person‐years were corrected by removing prevalent cases of parkinsonism.^20^ We computed age‐, sex‐, and decade‐specific incidence rates for all types of parkinsonism and for DIP. Incidence rates were directly standardized by age to the total U.S. population from the 1990 decennial census (midpoint of the 30‐year period) when rates for all ages combined were compared.^21^ Overall rates were also adjusted for age and sex using the same standard to facilitate comparison with findings from other populations.

We performed statistical testing of the time trends for DIP using negative binomial regression models.^22^ Negative binomial regression was used instead of Poisson regression because we had a number of zero counts and larger variance in some models.^22^ The unit of observation was the incidence rate in a single calendar year (directly standardized by age to the total 1990 U.S. population).^21^ We calculated relative risks (RRs) and the corresponding 95% CIs to measure the average change in the incidence rate over 10 calendar years. All statistical testing was done at the conventional two‐tailed alpha level of 0.05. For the analyses, we used SAS software (version 9.3; SAS Institute Inc., Cary, NC).

## Results

### Incidence and Time Trends

**Figure 1:**
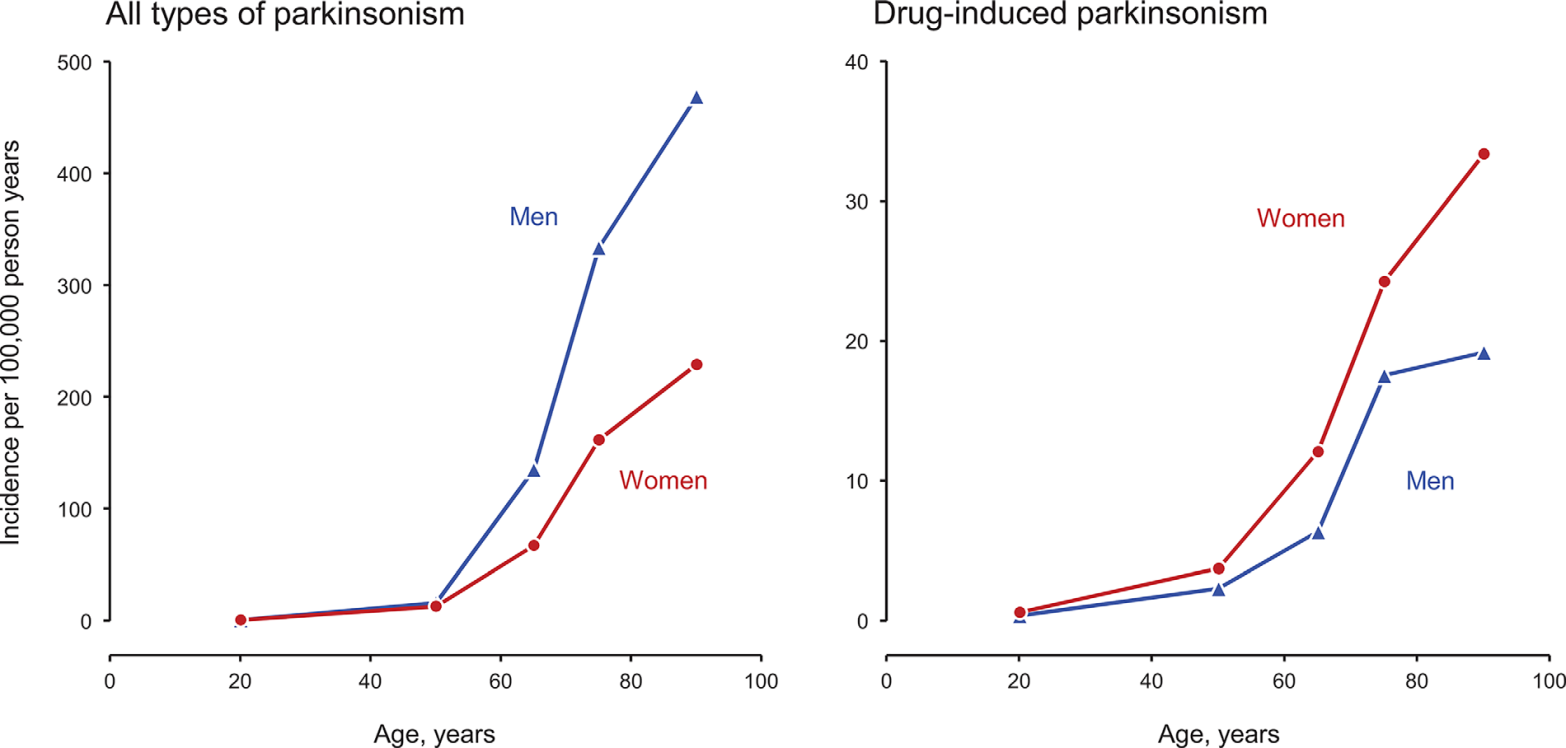
Average annual age‐ and sex‐specific incidence rates for the 30‐year period from 1976 to 2005 in men and women separately for all types of parkinsonism (left panel) and drug‐induced parkinsonism (right panel). The y‐axis scale is different in the two panels.

We identified 906 persons with onset of parkinsonism between January 1, 1976 and December 31, 2005. Of these patients, 108 (11.9%) had DIP. Supporting Table 1 shows the age‐, sex‐, and calendar year‐specific person‐year denominators used to compute the incidence rates. Figure 1 shows the age‐ and sex‐specific incidence rates of all types of parkinsonism (left panel) and DIP (right panel) over the full 30‐year period. Table 1 shows the age‐ and sex‐specific incidence rates for all types of parkinsonism and for DIP in three decades (1976–1985, 1986–1995, and 1996–2005). The average annual incidence rate of DIP over 30 years was 3.3 per 100,000 person‐years overall (108 patients), 2.1 in men (33 patients), and 4.3 in women (75 patients). In the youngest age group (0–39 years), DIP was the most common type of parkinsonism, accounting for 11 of 15 cases (73.3%).

**Table 1:** Incidence rates of all types of parkinsonism and DIP in Olmsted County, Minnesota, from 1976 to 2005 (new cases per 100,000 person‐years)^a^

		Age Group (Years)						
Group	Decade	0–39	40–59	60–69	70–79	80–99	All ages	All ages, standardized^b^
All types of parkinsonism								
Men	1976–1985	0.3 (1)	18.9 (15)	122.5 (30)	237.2 (33)	392.5 (24)	23.5 (103)	38.9
	1986–1995	0.8 (3)	14.1 (15)	120.3 (36)	307.7 (56)	379.4 (31)	27.1 (141)	41.7
	1996–2005	0.8 (3)	15.9 (25)	154.1 (61)	407.1 (101)	571.0 (67)	41.7 (257)	55.9
	All years	0.7 (7)	16.1 (55)	135.1 (127)	333.8 (190)	468.9 (122)	31.8 (501)	47.2
Women	1976–1985	1.4 (5)	15.3 (13)	91.3 (27)	154.9 (36)	221.1 (35)	23.1 (116)	26.8
	1986–1995	0.8 (3)	7.8 (9)	46.8 (16)	166.7 (45)	169.1 (36)	19.0 (109)	20.3
	1996–2005	0.0 (0)	15.2 (26)	66.6 (29)	162.1 (52)	283.6 (73)	27.0 (180)	26.0
	All years	0.7 (8)	12.9 (48)	67.1 (72)	161.6 (133)	229.1 (144)	23.2 (405)	24.4
Men and women	1976–1985	0.9 (6)	17.0 (28)	105.4 (57)	185.7 (69)	268.9 (59)	23.3 (219)	31.1
	1986–1995	0.8 (6)	10.8 (24)	81.1 (52)	223.5 (101)	227.4 (67)	22.9 (250)	28.7
	1996–2005	0.4 (3)	15.5 (51)	108.3 (90)	268.9 (153)	373.6 (140)	34.0 (437)	38.4
	All years	0.7 (15)	14.4 (103)	98.9 (199)	232.0 (323)	299.3 (266)	27.3 (906)	33.3
DIP								
Men	1976–1985	0.3 (1)	3.8 (3)	16.3 (4)	14.4 (2)	16.4 (1)	2.5 (11)	3.6
	1986–1995	0.6 (2)	0.9 (1)	3.3 (1)	27.5 (5)	24.5 (2)	2.1 (11)	3.1
	1996–2005	0.3 (1)	2.6 (4)	2.5 (1)	12.1 (3)	17.0 (2)	1.8 (11)	2.1
	All years	0.4 (4)	2.3 (8)	6.4 (6)	17.6 (10)	19.2 (5)	2.1 (33)	2.8
Women	1976–1985	1.4 (5)	7.0 (6)	10.1 (3)	43.0 (10)	44.2 (7)	6.2 (31)	6.9
	1986–1995	0.5 (2)	1.7 (2)	11.7 (4)	25.9 (7)	37.6 (8)	4.0 (23)	4.2
	1996–2005	0.0 (0)	3.5 (6)	13.8 (6)	9.4 (3)	23.3 (6)	3.1 (21)	3.1
	All years	0.6 (7)	3.8 (14)	12.1 (13)	24.3 (20)	33.4 (21)	4.3 (75)	4.5
Men and women	1976–1985	0.9 (6)	5.5 (9)	12.9 (7)	32.3 (12)	36.5 (8)	4.5 (42)	5.7
	1986–1995	0.5 (4)	1.4 (3)	7.8 (5)	26.6 (12)	33.9 (10)	3.1 (34)	3.7
	1996–2005	0.1 (1)	3.0 (10)	8.4 (7)	10.5 (6)	21.3 (8)	2.5 (32)	2.6
	All years	0.5 (11)	3.1 (22)	9.4 (19)	21.5 (30)	29.3 (26)	3.3 (108)	3.8

a The number of observed incidence cases is reported in parentheses in the table. Incidence rates were calculated by dividing the number of observed incidence cases by the corresponding person‐year denominators as listed in Supporting Table 1. We did not report CIs because the study covered the target population completely (no sampling was involved). The findings for parkinsonism of all types are reproduced here from another publication to facilitate comparisons with the findings for DIP.^10^

b The incidence rates were directly standardized by age to the total 1990 U.S. population. The incidence rates directly standardized by age and sex to the total 1990 U.S. population were 31.4 for 1976–1985, 28.9 for 1986–1995, 37.9 for 1996–2005, and 33.5 for the full 30 years for parkinsonism, and they were 5.6 for 1976–1985, 3.7 for 1986–1995, 2.6 for 1996–2005, and 3.8 for the full 30 years for DIP.

**Figure 2:**
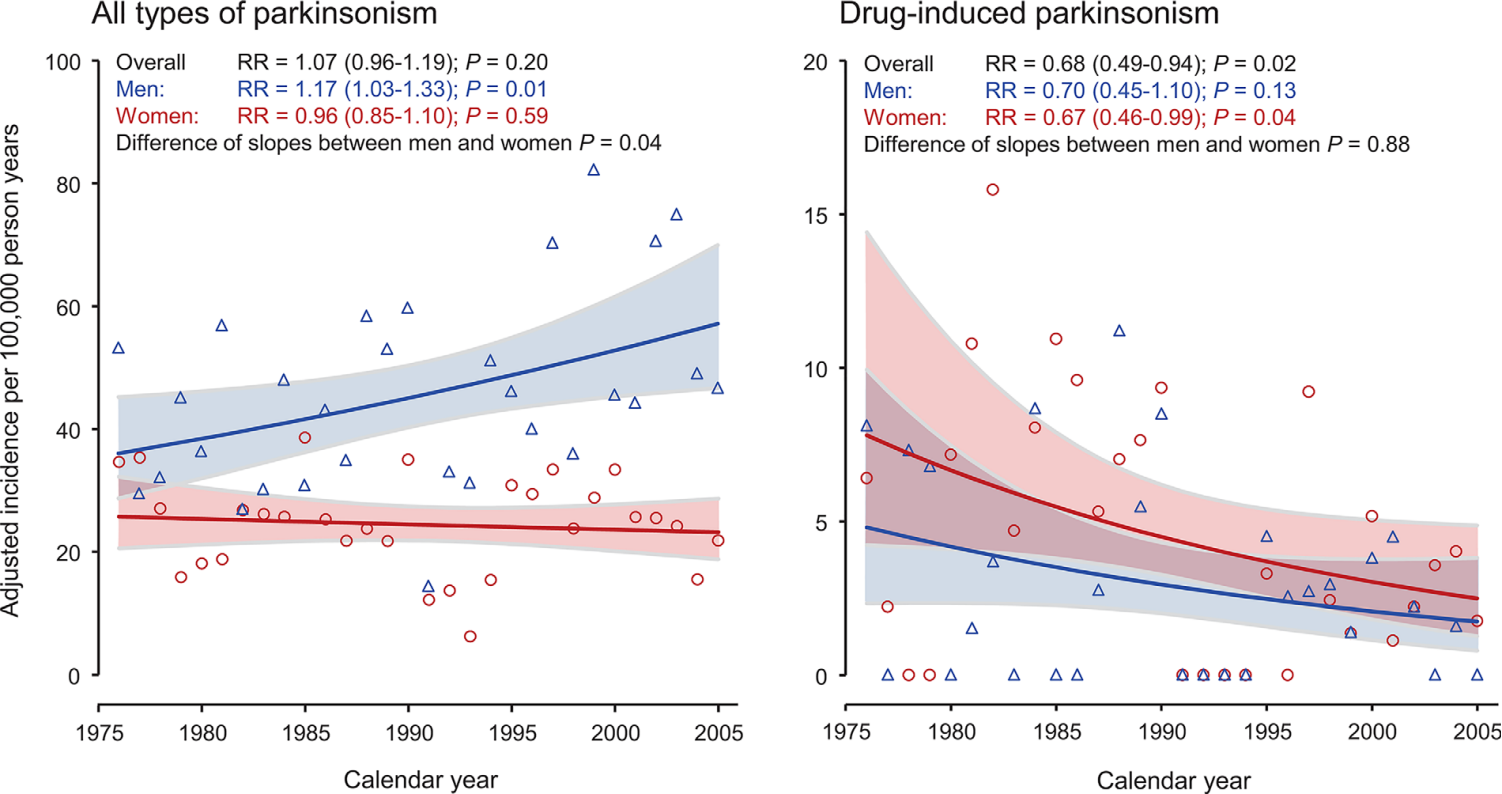
Incidence rate curves across calendar years for men and women estimated using single‐year data points and negative binomial regression for all types of parkinsonism (left panel) and drug‐induced parkinsonism (right panel). The y‐axis scale is different in the two panels. RR refers to the average change over 10 years.

Figure 2 shows the incidence rates estimated using single‐calendar‐year data points (directly age standardized to the total 1990 U.S. population) and negative binomial regression in men and women separately for all types of parkinsonism (left panel) and DIP (right panel). The incidence rates for parkinsonism were higher in men than women across the three decades, whereas the incidence rates for DIP were higher in women than men. We observed an overall decrease in the incidence of DIP of 32.0% per decade (RR = 0.68; 95% CI: 0.49–0.94) and 68.6% over the entire 30 years. Although the incidence of DIP decreased similarly over time in both men (RR = 0.70 per decade; 95% CI: 0.45–1.10) and women (RR = 0.67 per decade; 95% CI: 0.46–0.99), only the decrease in women was statistically significant.

### Clinical Characteristics

Table 2 shows the clinical characteristics of the 108 incident cases of DIP. Median age at onset of DIP was 70.9 years (interquartile range [IQR]: 54.4–79.7). Parkinsonism was tremor predominant in 57.4% of patients and akinetic rigid in 42.6%. Clinical features were asymmetric in 17.6%. Levodopa use was reported in only 12 patients who manifested more‐severe symptoms and for whom the clinician suspected a primary parkinsonian disorder. However, only 2 patients showed some response to treatment (Table 2, footnote c). Supporting Table 2 shows the distribution of the 108 patients by primary indication for treatment, specific drug considered responsible for DIP, sex, and decade of study. Typical antipsychotic medications were the drugs most frequently associated with DIP. A total of 64 patients (59.3%) were treated for schizophrenia and psychosis, 18 (16.7%) for severe mood disorders, 13 (12.0%) for severe dementia with agitation, 8 (7.4%) for chronic nausea, and 5 (4.6%) for other indications. Alternative groupings of patients can be derived from the complete display of data in Supporting Table 2 (e.g., grouping by class of drugs).

**Table 2:** Clinical characteristics of the 108 incident cases of DIP in Olmsted County, Minnesota, from 1976 to 2005

	Men (n = 33)		Women (n = 75)		*P* Value^a^	Men and Women (n = 108)	
Characteristic	n	%	n	%	Men vs. Women	n	%
Age of onset, years (median, IQR)^b^	68.4	51.1–77.4	73.7	58.1–83.7	0.10	70.9	54.4–79.7
Predominant symptom							
Rest tremor	20	60.6	42	56.0	0.68	62	57.4
Akinetic rigid	13	39.4	33	44.0		46	42.6
Asymmetry							
With asymmetry	6	18.2	13	17.3	0.99	19	17.6
Without asymmetry	27	81.8	62	82.7		89	82.4
Treatment							
Levodopa ever treated^c^	3	9.1	9	12.0	0.75	12	11.1
Never treated	30	90.9	66	88.0		96	88.9

a For the continuous variable (age of onset), the *P* value is from the Wilcoxon rank‐sum test for a difference of medians. For the categorical variables (rest tremor, asymmetry, and levodopa treatment), the *P* value is from Fisher's exact tests for a difference of proportions.

b Age of onset is reported as the median and IQR given as 25th and 75th percentiles.

c Only 2 of the 9 women and none of the 3 men treated with levodopa showed some response.

**Figure 3:**
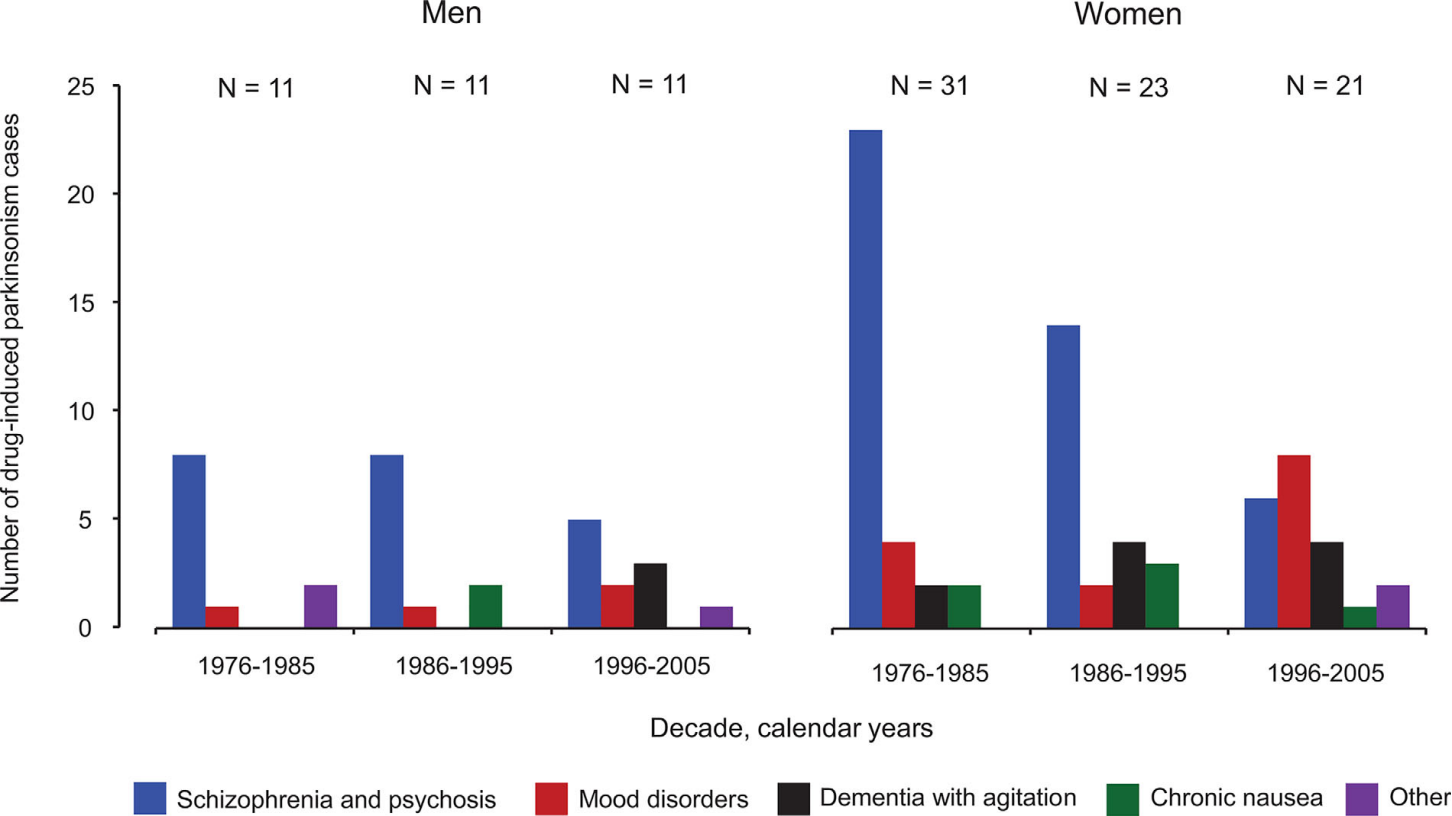
Distribution of incident cases of drug‐induced parkinsonism by decade of study and primary indication for treatment in men and women separately.

Figure 3 shows graphically the distribution of patients with incident DIP by primary indication for treatment and decade of study in men and women separately (aggregated data from Supporting Table 2). Although the numbers were small, the number of patients who experienced DIP because of treatment for mood disorders or dementia with agitation increased over time.

## Discussion

Our study suggests that DIP is a relatively rare type of parkinsonism compared with other neurodegenerative types in persons aged 40 years or older. Our findings for parkinsonism of all types, and specifically for PD, were reported elsewhere.^10^ The most common drugs associated with the development of DIP were typical antipsychotics. By contrast, we found an infrequent association with more recent atypical antipsychotics. We found that other drugs, such as motility agents and antidepressants, were also associated with DIP. The incidence of DIP increased with older age, and was higher in women than men at all ages. Most cases of DIP were rest‐tremor predominant and symmetric; however, 42.6% of the patients had the akinetic rigid subtype. Interestingly, the symptoms were asymmetric at onset in 17.6% of patients.

DIP was first described in the early 1950s after the introduction of reserpine and chlorpromazine.^23^ The discovery of the role of dopamine blockers as possible causes of DIP contributed to understanding the role of dopamine in the pathophysiology of PD.^24^ In our study, we found a 68.6% reduction in the incidence of DIP over a 30‐year period from 1976 to 2005. Paralleling this trend was a decrease in the cases of DIP attributed to the use of typical antipsychotic drugs for schizophrenia and psychosis. Our study also showed the rare association of DIP with atypical antipsychotics, which were introduced more recently. Putting these findings together, it appears that DIP has become much less frequent as atypical antipsychotics began to substitute for typical antipsychotics in psychiatric practice.

Interestingly, the reversal of DIP does not correspond to the absence of pathology; in fact, dopamine‐blocking agents can unmask preclinical PD. A recent study showed that 6 of 7 patients with DIP had pathological findings compatible with an underlying PD.^25^ The clinical characteristics of DIP have been described as variable; however, it has been suggested that the symmetric akinetic‐rigid subtype is the more common form.^26^ By contrast, our findings suggest a slightly higher frequency of the rest‐tremor–predominant form, and some patients manifested asymmetry of symptoms at onset.

In our population‐based study, the average annual incidence rate, age and sex standardized to the total 1990 US population, was 3.8 per 100,000 person‐years for DIP compared to 33.5 for all types of parkinsonism. Thus, DIP may be considered a relatively infrequent type of parkinsonism. However, DIP was the most common type of parkinsonism in persons 0 to 39 years old. When considering all ages combined for the years 1991 to 2005, DIP was the fifth‐most common subtype of parkinsonism in our population, following PD, unspecified parkinsonism, dementia with Lewy bodies, and PD dementia.^3^ This pattern is in contrast with some past prevalence studies in which DIP was the second‐most common subtype of parkinsonism after PD.^26^, ^27^, ^28^, ^29^ In addition, some studies showed a high risk of DIP among patients using neuroleptics.^27^, ^30^ A study in northern Italy showed that 36% of persons who received neuroleptics on a regular basis for 6 months or longer within the 5‐year period preceding a prevalence study developed parkinsonism.^27^ A study in the United States (Medicare Current Beneficiary Survey) showed a 94% increased risk of parkinsonism in persons receiving a neuroleptic drug (odds ratio = 1.94; *P* < 0.01).^30^ However, differences in case definition, study design, calendar years of the study, and drug use in different countries may account for the large variability of the results.

Older age, being a woman, genetic variants, preexisting movement disorders, and cigarette smoking have been identified as risk factors for DIP.^26^, ^31^ Our study confirmed that DIP was more common in women than men, although, in contrast, PD and the other neurodegenerative types of parkinsonism were less common in women than men. We postulate that genetic, endocrine, behavioral, or social and cultural differences may explain these sex differences; however, the underlying mechanisms remain unknown.^32^, ^33^, ^34^, ^35^

Our study also confirmed the increasing risk of DIP with older age (Fig. 1, right panel). This increase in risk of DIP may reflect the age‐related decline in number and integrity of dopaminergic neurons^36^; however, this decline may be subtle and difficult to detect by imaging.^37^ The increase in risk of DIP with age may also reflect a higher use of dopamine‐blocking or dopamine‐depleting drugs in older subjects in recent years. For example, there has been an increase over time in the use of antipsychotic drugs for depression resistant to other medications especially in older patients.^38^, ^39^ In addition, although not supported by published guidelines, antipsychotic drugs are used in the elderly population for dementia with agitation, confusion and delirium, and sometimes even for generalized anxiety disorders, especially in long‐term residential facilities.^40^ Finally, the increase over time in the use of palliative care for the elderly may have caused an increased use of antipsychotic drugs.

Our study has a number of strengths. First, taking advantage of the medical records‐linkage system of the REP, we studied a large, well‐defined population (3,318,819 person‐years overall).^11^, ^12^, ^13^, ^14^ Second, our patients were followed for a number of years after the diagnosis of parkinsonism through the records‐linkage system, thus reducing the risk of misclassification by type.^3^, ^8^ Third, all of the cases were adjudicated by a movement disorders specialist at the time of medical records abstraction to reduce differences in the diagnostic criteria over time or across the different specialists. Fourth, all of the medical facilities in Olmsted County are included in the REP, and it is unlikely that a person living in the county would have been seen only at a medical facility outside of the system.^11^, ^12^, ^13^, ^14^ In addition, the Olmsted County population is stable, especially for persons aged 65 years or older, and there were no changes in the medical facilities caring for patients with parkinsonism during the study period.^11^

Our study also has a number of limitations. First, parkinsonism may not have been recognized or diagnosed in our general population. For example, some mild symptoms related to DIP may not have been described in the medical records because they were considered an inevitable consequence of the treatment with certain drugs (typical antipsychotics) resulting in an under‐reporting of DIP. To reduce the possibility of a delayed diagnosis of parkinsonism, we collected data for an additional 5 years after the incidence period (2006–2010). This allowed us to appropriately retro date the time of onset of symptoms when needed. However, this technique should have played a minor role for the detection of DIP because of the acute or subacute nature of the symptoms.

Second, the diagnoses obtained through review of historical medical records may be unreliable. Our small reliability study showed adequate agreement between the two neurologists for presence or absence of parkinsonism.^3^ However, we did not conduct a reliability study focusing specifically on DIP. Third, because neurological practices and diagnostic criteria changed over time, some patients had more complete diagnostic information available in their records than others. In addition, the classification of patients in a single clinical subtype involved clinical judgment (e.g., patients affected by parkinsonism with concurrent autonomic dysfunction, cognitive disorders, and use of neuroleptics). However, the adjudication of all patients by two movement disorder specialists (R.S. and J.H.B.) should have attenuated these possible differences. Fourth, our series of 108 incident patients with DIP was relatively small to conduct analyses of time trends specific for individual drugs or for specific clinical characteristics (e.g., akinetic rigid‐ vs. tremor‐predominant forms). Finally, we did not study cases of DIP associated with drugs without a recognized dopamine action because the role of these other drugs in causing parkinsonism remains uncertain.

## Conclusion

Our study documented a 68.6% reduction in the incidence rate of DIP over the 30 years from 1976 to 2005 that was similar in men and women. Nearly all cases of DIP were associated with the use of typical antipsychotics. Our findings suggest that the transition to the use of newer atypical antipsychotic over the 30 years of the study was the primary reason for the decline in DIP incidence.

## Author Roles

(1) Research Project: A. Conception, B. Organization, C. Execution; (2) Statistical Analysis: A. Design, B. Execution, C. Review and Critique; (3) Manuscript Preparation: A. Writing of the First Draft, B. Review and Critique.

R.S.: 1A, 1B, 1C, 2C, 3A, 3B

B.R.G.: 1B, 2A, 2B, 3B

J.H.B.: 1B, 1C, 3B

J.E.A.: 1C, 3B

M.M.M.: 1C, 3B

W.A.R.: 1A, 1B, 2A, 2C, 3B

## Financial Disclosures

Nothing to report.

## Supporting information

Additional Supporting Information may be found in the online version of this article at the publisher's web‐site.

Supplementary Information Table 1.

Supplementary Information Table 2.
