# Supplementary material for: Incidence and time trends of drug‐induced parkinsonism: A 30‐year population‐based study
Source: Mov Disord. 2016 Oct 25;32(2):227–34. doi: 10.1002/mds.26839 (PMC5318251; doi:10.1002/mds.26839)
Supplement: Supplementary file 1 — Supplementary Information Table 1. [file MDS-32-227-s001.docx]

| **SUPPORTING TABLE 1.** Person-year denominators used to compute the incidence rates by age, sex, and calendar year (decade). | | | | | | | |
| --- | --- | --- | --- | --- | --- | --- | --- |
|  |  | **Age group** | | | | | |
| **Group** | **Decade** | **0-39 y** | **40-59 y** | **60-69 y** | **70-79 y** | **80-99 y** | **All ages** |
| Men | 1976-1985 | 314,404 | 79,569 | 24,498 | 13,910 | 6,114 | 438,495 |
|  | 1986-1995 | 357,438 | 106,293 | 29,930 | 18,200 | 8,170 | 520,031 |
|  | 1996-2005 | 383,706 | 156,767 | 39,586 | 24,809 | 11,734 | 616,602 |
|  | All years | 1,055,548 | 342,629 | 94,014 | 56,919 | 26,018 | 1,575,128 |
|  |  |  |  |  |  |  |  |
| Women | 1976-1985 | 348,937 | 85,196 | 29,562 | 23,245 | 15,827 | 502,767 |
|  | 1986-1995 | 375,551 | 115,608 | 34,210 | 26,992 | 21,290 | 573,651 |
|  | 1996-2005 | 394,553 | 171,371 | 43,528 | 32,080 | 25,741 | 667,273 |
|  | All years | 1,119,041 | 372,175 | 107,300 | 82,317 | 62,858 | 1,743,691 |
|  |  |  |  |  |  |  |  |
| Men and women | 1976-1985 | 663,341 | 164,765 | 54,060 | 37,155 | 21,941 | 941,262 |
|  | 1986-1995 | 732,989 | 221,901 | 64,140 | 45,192 | 29,460 | 1,093,682 |
|  | 1996-2005 | 778,259 | 328,138 | 83,114 | 56,889 | 37,475 | 1,283,875 |
|  | All years | 2,174,589 | 714,804 | 201,314 | 139,236 | 88,876 | 3,318,819 |
