# Supplementary material for: Incidence and time trends of drug‐induced parkinsonism: A 30‐year population‐based study
Source: Mov Disord. 2016 Oct 25;32(2):227–34. doi: 10.1002/mds.26839 (PMC5318251; doi:10.1002/mds.26839)
Supplement: Supplementary file 2 — Supplementary Information Table 2. [file MDS-32-227-s002.docx]

| **SUPPORTING TABLE 2**. Distribution of the 108 incident cases of drug-induced parkinsonism by primary indication for treatment, name of drug, sex, and decade of study. | | | | | | | | | | | | | |
| --- | --- | --- | --- | --- | --- | --- | --- | --- | --- | --- | --- | --- | --- |
|  | **Number of drug-induced parkinsonism cases** | | | | | | | | | | |  |  |
|  | **Men** |  |  |  | **Women** |  |  |  | **Men and women** | |  |  | **Total,**  **n (%)** |
| **Indication / medication** | **1976-1985** | **1986-1995** | **1996-2005** |  | **1976-1985** | **1986-1995** | **1996-2005** |  | **1976-1985** | **1986-1995** | **1996-2005** |  | **1976-2005** |
| **Schizophrenia and**  **psychosis** | **8** | **8** | **5** |  | **23** | **14** | **6** |  | **31** | **22** | **11** |  | **64 (59.3)** |
| Chlorpromazine | 0 | 0 | 0 |  | 0 | 1 | 0 |  | 0 | 1 | 0 |  | 1 |
| Fluphenazine | 0 | 2 | 0 |  | 4 | 1 | 0 |  | 4 | 3 | 0 |  | 7 |
| Haloperidol | 1 | 3 | 0 |  | 3 | 6 | 0 |  | 4 | 9 | 0 |  | 13 |
| Loxapine | 1 | 0 | 0 |  | 0 | 0 | 0 |  | 1 | 0 | 0 |  | 1 |
| Molindone | 0 | 0 | 0 |  | 0 | 1 | 0 |  | 0 | 1 | 0 |  | 1 |
| Olanzapine | 0 | 0 | 1 |  | 0 | 0 | 1 |  | 0 | 0 | 2 |  | 2 |
| Perphenazine | 2 | 1 | 2 |  | 4 | 0 | 1 |  | 6 | 1 | 3 |  | 10 |
| Phenothiazine | 0 | 0 | 0 |  | 3 | 0 | 0 |  | 3 | 0 | 0 |  | 3 |
| Risperidone | 0 | 0 | 0 |  | 0 | 0 | 1 |  | 0 | 0 | 1 |  | 1 |
| Risperidone +  Olanzapine | 0 | 0 | 1 |  | 0 | 0 | 1 |  | 0 | 0 | 2 |  | 2 |
| Thioridazine | 1 | 0 | 0 |  | 6 | 1 | 0 |  | 7 | 1 | 0 |  | 8 |
| Thiothixene | 0 | 0 | 0 |  | 0 | 0 | 1 |  | 0 | 0 | 1 |  | 1 |
| Trifluoperazine | 3 | 2 | 1 |  | 2 | 4 | 1 |  | 5 | 6 | 2 |  | 13 |
| Unknown | 0 | 0 | 0 |  | 1 | 0 | 0 |  | 1 | 0 | 0 |  | 1 |
| **Mood disorders** | **1** | **1** | **2** |  | **4** | **2** | **8** |  | **5** | **3** | **10** |  | **18 (16.7)** |
| Chlorpromazine | 0 | 0 | 0 |  | 1 | 0 | 0 |  | 1 | 0 | 0 |  | 1 |
| Citalopram | 0 | 0 | 0 |  | 0 | 0 | 1 |  | 0 | 0 | 1 |  | 1 |
| Diazepam | 1 | 0 | 0 |  | 0 | 0 | 0 |  | 1 | 0 | 0 |  | 1 |
| Fluoxetine | 0 | 0 | 0 |  | 0 | 1 | 0 |  | 0 | 1 | 0 |  | 1 |
| Fluphenazine +  Amytriptiline | 0 | 0 | 0 |  | 0 | 0 | 1 |  | 0 | 0 | 1 |  | 1 |
| Haloperidol | 0 | 0 | 1 |  | 0 | 0 | 0 |  | 0 | 0 | 1 |  | 1 |
| **SUPPORTING TABLE 2**. Continued. | | | | | | | | | | | | | |
|  | **Number of drug-induced parkinsonism cases** | | | | | | | | | | | | |
|  | **Men** | | |  | **Women** | | |  | **Men and women** | | |  | **Total,**  **N (%)** |
| **Indication / medication** | **1976-1985** | **1986-1995** | **1996-2005** |  | **1976-1985** | **1986-1995** | **1996-2005** |  | **1976-1985** | **1986-1995** | **1996-2005** |  | **1976-2005** |
| Perphenazine | 0 | 0 | 0 |  | 0 | 1 | 1 |  | 0 | 1 | 1 |  | 2 |
| Phenothiazine | 0 | 0 | 0 |  | 0 | 0 | 1 |  | 0 | 0 | 1 |  | 1 |
| Risperidone | 0 | 0 | 0 |  | 0 | 0 | 3 |  | 0 | 0 | 3 |  | 3 |
| Thioridazine | 0 | 1 | 1 |  | 2 | 0 | 1 |  | 2 | 1 | 2 |  | 5 |
| Triazolam | 0 | 0 | 0 |  | 1 | 0 | 0 |  | 1 | 0 | 0 |  | 1 |
| **Dementia with agitation** | **0** | **0** | **3** |  | **2** | **4** | **4** |  | **2** | **4** | **7** |  | **13 (12.0)** |
| Haloperidol | 0 | 0 | 0 |  | 0 | 0 | 1 |  | 0 | 0 | 1 |  | 1 |
| Olanzapine | 0 | 0 | 2 |  | 0 | 0 | 1 |  | 0 | 0 | 3 |  | 3 |
| Perphenazine | 0 | 0 | 0 |  | 0 | 1 | 0 |  | 0 | 1 | 0 |  | 1 |
| Risperidone | 0 | 0 | 1 |  | 0 | 0 | 2 |  | 0 | 0 | 3 |  | 3 |
| Thioridazine | 0 | 0 | 0 |  | 2 | 3 | 0 |  | 2 | 3 | 0 |  | 5 |
| **Chronic nausea** | **0** | **2** | **0** |  | **2** | **3** | **1** |  | **2** | **5** | **1** |  | **8 (7.4)** |
| Metoclopramide | 0 | 2 | 0 |  | 2 | 3 | 1 |  | 2 | 5 | 1 |  | 8 |
| **Other** | **2** | **0** | **1** |  | **0** | **0** | **2** |  | **2** | **0** | **3** |  | **5 (4.6)** |
| Aripiprazole^a^ | 0 | 0 | 1 |  | 0 | 0 | 0 |  | 0 | 0 | 1 |  | 1 |
| Diphenhydramine | 1 | 0 | 0 |  | 0 | 0 | 0 |  | 1 | 0 | 0 |  | 1 |
| Prochlorperazine | 0 | 0 | 0 |  | 0 | 0 | 1 |  | 0 | 0 | 1 |  | 1 |
| Procainamide | 1 | 0 | 0 |  | 0 | 0 | 0 |  | 1 | 0 | 0 |  | 1 |
| Unknown | 0 | 0 | 0 |  | 0 | 0 | 1 |  | 0 | 0 | 1 |  | 1 |
| **Total** | **11** | **11** | **11** |  | **31** | **23** | **21** |  | **42** | **34** | **32** |  | **108 (100.0)** |

^a^ Aripiprazole was included in the “Other” category because it is a third generation antipsychotic drug with atypical pharmacodynamics and pharmacokinetic characteristics.
